# Plate fixation versus flexible intramedullary nails for management of closed femoral shaft fractures in the pediatric population: A systematic review and meta-analysis of the adverse outcomes

**DOI:** 10.1177/18632521231190713

**Published:** 2023-08-28

**Authors:** Abhinav Singh, William Bierrum, Justin Wormald, Manoj Ramachandran, Gregory Firth, Deborah Eastwood

**Affiliations:** 1 Nuffield Department of Orthopaedics, Rheumatology and Musculoskeletal Sciences, University of Oxford, Oxford, UK; 2 Department of Orthopaedic Surgery, Imperial College NHS Healthcare Trust, London, UK; 3 Department of Acute Internal Medicine, University College London Hospital NHS Trust, London, UK; 4 Department of Orthopaedic Surgery, Barts Health NHS Trust, London, UK; 5 Department of Orthopaedic Surgery, Great Ormond Street Hospital, London, UK; 6 University College London, London, UK

**Keywords:** Children, femur fracture, plate fixation, intramedullary nail, meta-analysis

## Abstract

**Purpose:**

Fractures of the femoral diaphysis are associated with a risk of morbidity in children. Various fixation methods have been developed, but with only limited evidence to support their use. This systematic review assesses the evidence regarding clinical outcomes of closed femoral diaphyseal fractures in children treated with plate fixation or flexible intramedullary nails.

**Methods:**

A PROSPERO-registered, PRISMA-compliant systematic review and meta-analysis were conducted. MEDLINE, Embase, and Web of Science (WoS) databases were searched from inception to February 2023. Inclusion criteria included clinical studies reporting adverse outcomes following surgical treatment of pediatric closed femoral diaphyseal fractures using plate fixation and flexible intramedullary nails. The ROBINS-I and RoB 2 tools evaluated the risk of bias.

**Results:**

Thirteen papers (2 prospective randomized controlled trials and 11 retrospective cohorts) reported 805 closed diaphyseal femoral fractures in 801 children (559 males, 242 females). There were 360 plate fixations and 445 flexible intramedullary nails. Two cases of osteomyelitis and one nonunion were reported. Meta-analysis showed that plate fixation had a lower risk of soft tissue infection (relative risk 0.26 (95% confidence interval 0.07–0.92)). There was no difference in the following outcomes: malunion (relative risk 0.68 (95% confidence interval 0.32–1.44)); unplanned reoperation (relative risk 0.59 (95% confidence interval 0.31–1.14)), and leg-length difference (relative risk 1.58 (95% confidence interval 0.66–3.77)). The risk of bias was high in all studies.

**Conclusions:**

An analysis of 805 fractures with minimal differences in meta-analyses is considered high quality even when the quality of the evidence is low. The findings are limited by important flaws in the methodology in the published literature. Well-designed multicentre prospective studies using standardized core outcomes are required to advise treatment recommendations.

**Level of evidence:**

III.

## Introduction

Fractures of the femoral diaphysis (shaft) are the most common major pediatric injury treated by orthopedic surgeons.^1,2^ Their reported incidence ranges from 5.82 to 16.4 per 100,000 children and fractures occur most frequently in the summer.^3,4^ They are the leading cause for hospitalization in pediatric trauma patients and a significant cause of morbidity.^5^ These injuries often require prolonged immobilization and/or surgery which contributes to significant psychological stresses on the patients and their parents/guardian. By 14 years of age, males are 4.7 times more likely than females to have had a femoral shaft fracture.^6^

Despite the impact of this injury, definitive treatment in patients aged 4–12 years and <50 kg continues to be a controversial area. Historically, there has been a preference toward a particular “in vogue” surgical treatment (flexible intramedullary nails (FIN) in the 1990s or plate fixation (PF) in the 2000s), demonstrated in scoping reviews.^5,7^ In the last 15 years, FINs have become popular again, despite the lack of high-quality evidence to support their use.^1,8^ Importantly, the lack of evidence has resulted in ongoing regional practice variation in the management of this injury.^3^

This systematic review aimed to answer the question: in children with closed diaphyseal femoral fractures, what is the risk of negative outcomes following fixation with plates (PF) versus flexible intramedullary nails (FIN)? Secondary aims were to inform practice surrounding the consenting of patients and research in the future.

## Methods

We used the methodology outlined in the Cochrane Handbook for Systematic Review of Interventions.^9^ The review is reported in accordance with the Preferred Reporting Items for Systematic Reviews and Meta-Analyses (PRISMA) statement^10^ and the meta-analysis of observational studies in epidemiology (MOOSE) guidelines.^11^ An a priori protocol was registered prospectively on the PROSPERO international register of systematic reviews (CRD42020193281).^12^

**Figure 1.**
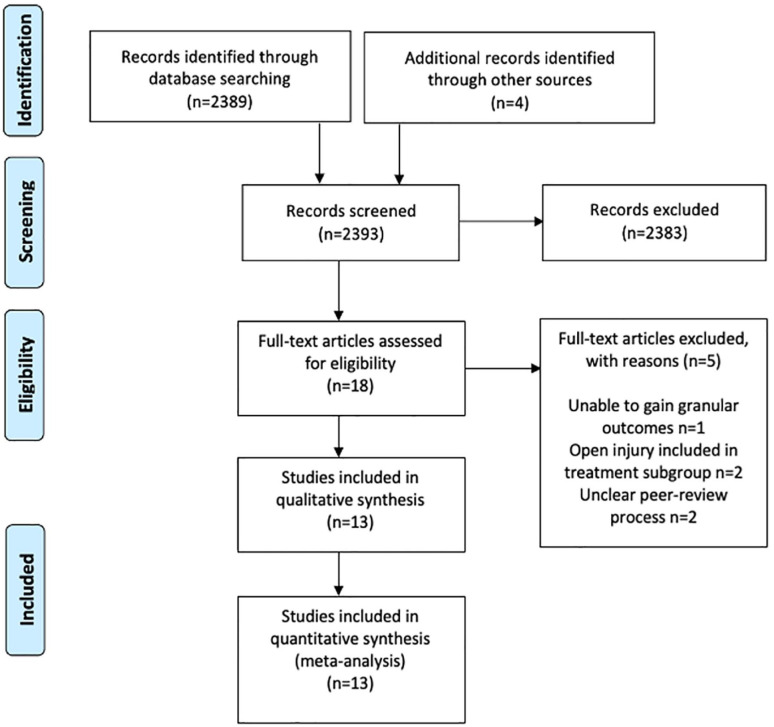
PRISMA flowchart of study attrition.

MEDLINE, Embase, and Web of Science (WoS) were searched from the outset to February 2023 using MeSH terms and free-text strategies (Figure 1, Supplemental Appendix 1). Two authors (A.S. and W.B.) searched independently, with medical librarian support. No language limits were applied, and the reference lists of included articles were hand-searched to identify additional publications. The gray literature was searched using Google Scholar. To ensure the best possible direct comparison between the two groups, only studies reporting adverse outcomes following PF and FIN in children with a closed femoral fracture were included. The fracture was the unit of analysis.

The co-primary outcomes were malunion and nonunion. The definition of a malunion was a deformity of 15–30° in the sagittal plane and 10° in the frontal plane requiring operative intervention. Nonunion was an arrest or absence of healing on serial radiographs, 6 months after primary management. The use of the term (malunion/nonunion) within the included studies was deemed adequate. Secondary outcomes were infection (osteomyelitis (OM) and/or soft tissue infection), unplanned reoperation, leg-length difference (>1 cm), time to union (weeks), length of stay (days), operative duration (minutes), and blood loss (ml).

For patient demographics, treatments and outcome data, descriptive analyses were performed. When appropriate, individual study incidences of adverse events were pooled, and meta-analysis was performed using RevMan5 (The Cochrane Collaboration, Copenhagen, Denmark). Relative risks (RRs) and 95% confidence intervals (CIs) were calculated using the Cochran–Mantel–Haenszel test. A random-effects model was used because of the anticipated study heterogeneity. Forest plots were used to display results. Publication bias was viewed with funnel plots.^13,14^ The *I*^2^ statistic measured heterogeneity.^15^

## Results

In total, 2389 articles were identified using our search strategy. Thirteen studies (11 retrospective cohorts; 2 prospective randomized controlled trials) reporting 805 closed femoral fractures in 801 children were eligible for inclusion (Table 1).^1,16–27^ Studies were from the United Kingdom, United States, Israel, India, China, and Pakistan. There were 559 males (69.8%) and 242 females (30.2%) with the reported mean ages in the included studies ranging from 5.9 to 10.6 years. The average patient weight was similar in both groups (30.6 kg in PF; 28.4 kg in FIN). Mean follow-up was 27.5 months (range 3–63.6) and of 805 fractures, 360 (44.7%) were managed with PF, and 445 (55.3%) with FIN (Table 2).

**Table 1.** Characteristics of included studies.

Study ID and author	Title	Year	Location	Methods	Patients	Fractures	Mean age—plates (years)	Mean age—nails (years)	Male	Female	Mean follow-up—plates (months)	Mean follow-up—nails (months)
1. Milligan et al.^16^	Elastic nail fixation versus plate fixation of pediatric femoral fractures in school age patients—A retrospective observational study	2020	United Kingdom	Retrospective case series	28	28	7.7	9.7	21	7	63.6	63.6
2. Wang et al.^17^	Comparison of efficacy between internal fixation of minimally invasive elastic stable intramedullary nail and plate in the treatment of pediatric femoral shaft fracture	2019	China	Retrospective case series	120	120	6.6	10.4	74	46	–	–
3. Chen et al.^18^	Submuscular plates versus flexible nails in preadolescent diaphyseal femur fractures	2018	United States	Retrospective case series	58	58	8.1	7.3	43	15	21	22
4. Xu et al.^19^	Titanium elastic nailing versus locking compression plating in school-aged pediatric subtrochanteric femur fractures	2018	China	Retrospective case series	67	67	9.4	6.8	40	27	28.5	28.5
5. Luo et al.^20^	Elastic stable titanium flexible intramedullary nails versus plates in treating low grade comminuted femur shaft fractures in children	2019	China	Retrospective case series	51	51	5.9	5.9	35	16	29	29
6. Sutphen et al.^21^	Pediatric diaphyseal femur fractures: Submuscular plating compared with intramedullary nailing	2016	United States	Retrospective case series	96	96	10.6	10.6	72	24	48	48
7. Allen et al.^22^	Titanium elastic nailing has superior value to plate fixation of midshaft femur fractures in children 5 to 11 years	2018	United States	Retrospective case series	63	65	8	9	43	20	–	–
8. Li et al.^23^	Comparison of titanium elastic nail and plate fixation of pediatric subtrochanteric femur fractures	2013	United States	Retrospective case series	54	54	8.4	7.9	42	12	21.6	21.6
9. Sela et al.^1^	Pediatric femoral shaft fractures: treatment strategies according to age—13 years of experience in one medical center	2013	Israel	Retrospective case series	24	24	10.5	9.7	13	11	–	–
10. Ahmed et al.^24^	Comparison of treatment with Titanium Elastic Nail versus AO—Dynamic compression plate for femoral shaft fractures in children	2016	Pakistan	Retrospective case series	60	60	8.9	8.8	48	12	24	24
11. James et al.^25^	Elastic stable intramedullary nailing versus submuscular plating in pediatric diaphyseal femur fractures: a randomized controlled trial among children in South India	2022	India	Prospective randomized controlled trial	40	40	10.2	9.5	26	14	24	24
12. Caglar et al.^26^	Comparison of compression plate and flexible intramedullary nail fixation in pediatric femoral shaft fractures	2006	Turkey	Retrospective case series	38	40	8	8.1	24	14	–	–
13. Hayat et al.^27^	Comparison of submuscular locking plate and titanium elastic nail in children with fracture midshaft of the femur	2021	Pakistan	Prospective randomized controlled trial	102	102	9.3	8.8	78	24	3	3

**Table 2.** Injury characteristics and management techniques.

Study ID and author	Fracture total	Anatomical location			Fracture type			Surgical procedure		
		Proximal/subtrochanteric	Mid diaphysis	Distal diaphysis	Classification	Length stable	Length unstable	Plates	Nails	Total
1. Milligan et al.^16^	28	–	28	–	–	–	–	14	14	28
2. Wang et al.^17^	120	120	–	–	Seinsheimer	–	–	60	60	120
3. Chen et al.^18^	58	–	58	–	–	16	42	30	28	58
4. Xu et al.^19^	67	67	–	–	Pombo & Shilt	48	19	28	39	67
5. Luo et al.^20^	51	12	33	6	Winquist	16	42	22	29	51
6. Sutphen et al.^21^	96	15	64	17	–	44	52	35	61	96
7. Allen et al.^22^	65	–	65	–	AO-OTA	44	21	15	50	65
8. Li et al.^23^	54	54	–	–	–	10	44	29	25	54
9. Sela et al.^1^	24	–	24	–	–	16	8	3	21	24
10. Ahmed et al.^24^	60	11	42	7	–	–	–	31	29	60
11. James et al.^25^	40	10	22	8	AO	28	13	20	20	40
12. Caglar et al.^26^	40	–	40	–	–	20	20	22	18	40
13. Hayat et al.^27^	102	–	102	–	–	–	–	51	51	102
Total (*n*)	805	289	478	38	–	242	261	360	445	805
%		36	59.3	4.7				44.7	55.3	

The co-primary outcomes (malunion and nonunion) were reported in all studies (Table 3). There were two cases of osteomyelitis (PF) and one nonunion (FIN), due to the low event rate meta-analysis of these subgroups was not possible. With respect to the secondary outcomes, infection (of the bone or the soft tissues) was reported in all studies (Table 3).^1,16–27^ Twelve studies^1,16,18–27^ reported leg-length difference and 11 studies^16–27^ reported unplanned reoperations (Table 4). These secondary outcomes were pooled and are expressed as summary statistics. Eight or fewer studies reported on time to union,^16,17,20,21,23,25–27^ length of stay,^1,16,17,19,20,23^ operative duration,^17,19,20,22,25–27^ and blood loss.^17,19,20,22^ Due to the sparsity of reporting around these outcomes, descriptive analysis was used (Table 4).

**Table 3.** Primary adverse outcomes.

Study ID and author	Study total	Nonunion (N)				Malunion (M)			
		Plate		Nails		Plate		Nails	
		*n*	N	*n*	N	*n*	M	*n*	M
1. Milligan et al.^16^	28	14	0	14	0	14	0	14	0
2. Wang et al.^17^	120	60	0	60	0	60	4	60	2
3. Chen et al.^18^	58	30	0	28	0	30	0	28	0
4. Xu et al.^19^	67	28	0	39	0	28	0	39	0
5. Luo et al.^20^	51	22	0	29	0	22	0	29	0
6. Sutphen et al.^21^	96	35	0	61	0	35	4	61	13
7. Allen et al.^22^	65	15	0	50	0	15	1	50	2
8. Li et al.^23^	54	29	0	25	0	29	1	25	4
9. Sela et al.^1^	24	3	0	21	0	3	0	21	0
10. Ahmed et al.^24^	60	31	0	29	0	31	0	29	0
11. James et al.^25^	40	20	0	20	0	20	0	20	1
12. Caglar et al.^26^	40	22	0	18	1	22	0	18	0
13. Hayat et al.^27^	102	51	0	51	0	51	0	51	0
Total (n)	805	360	0	445	1	360	10	445	22
Incidence (%)			0		0.2		2.8		5

**Table 4.** Secondary adverse outcomes.

Study ID and author	Total	Soft tissue infection (SI)				Osteomyelitis (OM)				Unplanned reoperation (R)				Leg-length difference (LLD)			
		Plate		Nail		Plate		Nails		Plate		Nails		Plate		Nails	
		*n*	SI	*n*	SI	*n*	OM	*n*	OM	*n*	R	*n*	R	*n*	LLD	*n*	LLD
1. Milligan et al.^16^	28	14	0	14	0	14	0	14	0	14	0	14	4	14	1	14	0
2. Wang et al.^17^	120	60	0	60	2	60	0	60	0	–	–	–	–	–	–	–	–
3. Chen et al.^18^	58	30	1	28	2	30	0	28	0	30	2	28	7	30	0	28	0
4. Xu et al.^19^	67	28	0	39	0	28	0	39	0	28	0	39	0	28	3	39	1
5. Luo et al.^20^	51	22	0	29	0	22	0	29	0	22	2	29	2	22	0	29	0
6. Sutphen et al.^21^	96	35	0	61	0	35	0	61	0	35	1	61	0	35	1	61	3
7. Allen et al.^22^	65	15	0	50	0	15	0	50	0	15	0	50	1	15	1	50	1
8. Li et al.^23^	54	29	0	25	1	29	0	25	0	29	1	25	2	29	2	25	0
9. Sela et al.^1^	24	3	0	21	0	3	0	21	0	–	–	–	–	3	0	21	3
10. Ahmed et al.^24^	60	31	0	29	1	31	0	29	0	31	0	29	0	31	0	29	0
11. James et al.^25^	40	20	0	20	0	20	1	20	0	20	1	20	4	20	1	20	2
12. Caglar et al.^26^	40	22	0	18	0	22	0	18	0	22	4	18	1	22	0	18	0
13. Hayat et al.^27^	102	51	0	51	3	51	1	51	0	51	0	51	0	51	0	51	0
Total (n)	805	360	1	445	9	360	2	445	0	297	11	364	21	300	9	385	10
Incidence (%)			0.3		2		0.6		0		3.7		5.8		3		2.6
Study ID & Author	Total	Time to union (weeks)				Length of stay (days)				Operation duration (min)				Blood loss (ml)			
		Plate		Nails		Plate		Nails		Plate		Nails		Plate		Nails	
1. Milligan et al.^16^	28	12		10		6.3		7.8		–		–		–		–	
2. Wang et al.^17^	120	11.5		9		15.3		6.8		142.4		69		142.2		61.3	
3. Chen et al.^18^	58	–		–		–		–		–		–		–		–	
4. Xu et al.^19^	67	–		–		6.5		5.7		98		41.2		70		8.2	
5. Luo et al.^20^	51	11.4		10		11		5.8		100.8		69.4		163.3		18.3	
6. Sutphen et al.^21^	96	6.2		8.1		–		–		–		–		–		–	
7. Allen et al.^22^	65	–		–		–		–		150		96		79		40.1	
8. Li et al.^23^	54	11.4		10.7		3.4		3.2		–		–		–		–	
9. Sela et al.^1^	24	–		–		–		–		–		–		–		–	
10. Ahmed et al.^24^	60	11		6		–		–		53.3		29.9		–		–	
11. James et al.^25^	40	10.2		9.5		5.4		4.4		104.7		99.9		–		–	
12. Caglar et al.^26^	40	20.6		17		–		–		100.3		84.5		–		–	
13. Hayat et al.^27^	102	–		–		–		–		–		–		–		–	
Mean		10.6		8.9		8.5		5.9		108.9		61.1		113.6		32	

**Figure 2.**
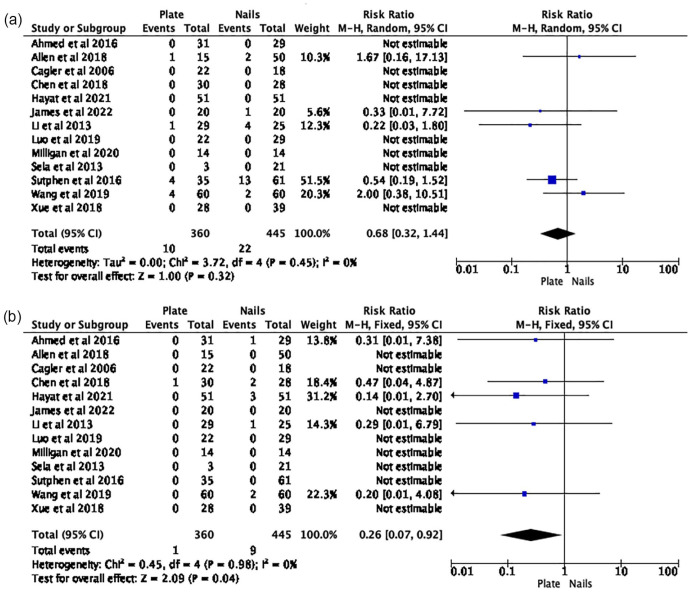
(a) Risk of malunion: plate fixation versus intramedullary nails. (b) Risk of soft tissue infection: plate fixation versus intramedullary nails.

**Figure 3.**
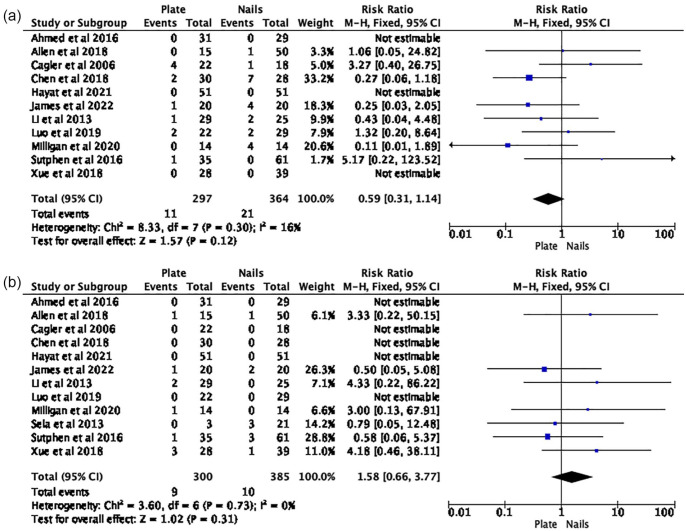
(a) Risk of unplanned reoperation: plate fixation versus intramedullary nails. (b) Risk of leg-length difference: plate fixation versus intramedullary nails.

The risk estimates from all 13 papers were amalgamated for the meta-analysis. A 74% reduction in the risk of soft tissue infection (RR 0.26 (95% CI 0.07–0.92)) was identified in PF group (Figure 2(b)). There was no difference in malunion (RR 0.68 (95% CI 0.32–1.44)) (Figure 2(a)); unplanned reoperation (RR 0.59 (95% CI 0.31–1.14)) (Figure 3(a)) and leg-length difference (RR 1.58 (95% CI 0.66–3.77)) (Figure 3(b)). Descriptive analysis showed a longer time to union, length of stay, operative duration, and blood loss for the PF group. When comparing PF versus FIN, soft tissue infection rate was 0.3% (*n* = 1) versus 2% (*n* = 9); time to union (weeks) 12 (range 6.2–20.6) versus 10.7 (range 8.1–17); length of stay (days) 8 (range 3.5–15.3) versus 5.6 (range 3.2–7.8); operation duration (minutes) 107.1 (range 98–150) versus 70 (range 41.2–99.9); and blood loss 113.6 (range 70–163.3) versus 32 (range 8.2–61.3).

As assessed by the ROBINS-I tool for non-randomized studies of intervention (Table 5),^28^ and because of the retrospective observational nature of these studies, there were few pre-published protocols. All studies were at moderate to serious risk of bias due also to confounding between treatment groups, attrition, and bias in reporting. The RoB 2 tool was used for randomized studies (Table 6),^29^ and revealed a high risk of bias due to underpowering, lack of prospective statistical analysis plan and missing outcome data. Most studies also lacked homogeneous definitions of outcomes (Tables 5 and 6).

**Table 5.** Study definitions of outcome measure and risk of bias assessment (non-randomized studies).

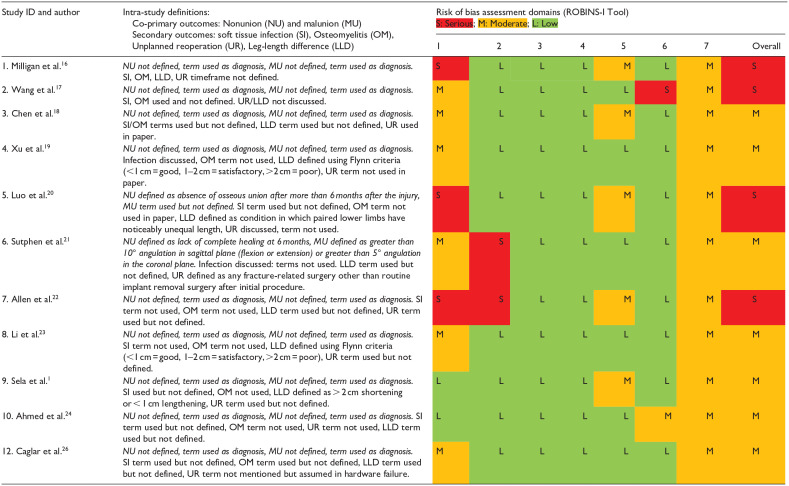

Risk of bias domains: Pre-intervention—(1) Confounding, (2) Selection bias; Intervention—(3) Bias in measurement classification of interventions; Post-intervention—(4) Bias due to deviations from intended interventions; (5) Bias due to missing data; (6) Bias in measurement of outcomes; (7) Bias in selection of the reported result.

**Table 6.** Study definitions of outcome measure and risk of bias assessment (randomized studies).

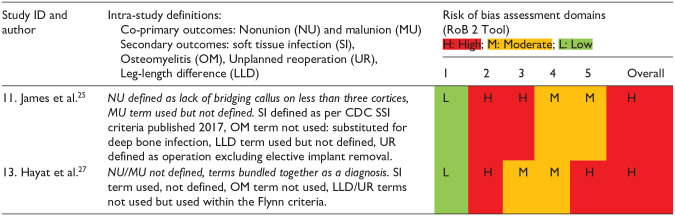

Bias domains: (1) Bias arising from randomization, (2) Bias due to deviation from intended intervention, (3) Bias due to missing outcome data, (4) Bias in measurement of the outcome, (5) Bias in selection of the reported result.

## Discussion

The anatomy of a child’s femur is fundamentally different to that of an adult. The presence of a thicker and more vascular periosteum means that the bone has a high healing and remodeling capacity. The pediatric femur is able to tolerate up to 15° of malrotation and 25° of angulation in any plane.^30,31^ In these patients, various operative strategies have been advocated to reduce the adverse consequences be they physical, social, psychological, and/or financial associated with lengthy periods of immobilization.^1^ As younger children (≤ 4 years) have better healing potential, non-surgical treatment (traction or spica cast) is first-line.^32^ In older children (5–12 years), surgical treatment is preferred to aid an earlier return to weightbearing/activity and to prevent prolonged periods of absence from school.^1,2^ Children > 12 years and/or weighing > 50 kg are treated with rigid intramedullary nails to balance mechanical stresses at the fracture site with diminishing remodeling potential.^2,8^

However, definitive management of closed femoral shaft fractures in patients aged 4–12 years and <50 kg continues to be a controversial area with marked regional differences. It is argued that, biomechanically, length stable (transverse) fractures would be more suitable for FIN^18^ whereas, PF may be superior for unstable (long oblique, comminuted) fractures.^16^ These variations in fracture patterns and the suitability of PF versus FIN in achieving healing without adverse outcomes continue to be debated. Notably, the American Academy of Orthopedic Surgeons (AAOS),^32^ the United Kingdom’s National Institute of Clinical Excellent (NICE)^33^ and a recent Cochrane review^34^ have all emphasized the poor quality of available evidence when making management recommendations. To address this ongoing debate, this systematic review’s methodology focuses on patients within a specific mean age range (5–12) and weight (<50 kg). It compares adverse outcomes following operative management (PF vs FIN) of closed pediatric femoral shaft fractures. Direct comparison meta-analysis of 805 fractures indicates a substantially reduced risk of soft tissue infection in the PF group, with no difference in malunion, unplanned reoperation and leg-length difference.

The literature already suggests that PF may confer advantages through earlier full weightbearing and time to fracture union however, the markedly reduced risk of infection in this group is an interesting finding. This could be secondary to advances in PF techniques which include stab incisions for submuscular plating compared to the large open approach.^2^ It could also be due to the fact that while FIN requires a smaller wound and offers a shorter hospital stay,^2^ it has a higher incidence of metalwork prominence which may irritate/ulcerate the soft tissue leading to a wound infection. This is of value to clinicians when organizing their follow-up protocols. While the marked cost difference of implants in favor of FIN (FIN—£138 for 2; PF (locking compression plate, LCP)—£260) may lead to its preferential usage in some units, this benefit must be carefully balanced against the cost implications of unplanned hospital admission and/or reoperation, which continues to rise within healthcare systems.^35^

The disadvantages of both techniques are also well-reported: with PF, longer operative time and increased blood loss, difficulty in plate removal, re-fracture following removal; and with FIN, longer time to weightbearing, higher rates of malunion and leg-length difference.^1,2,5^ Our descriptive analysis clearly demonstrates these disadvantages (Tables 4a/b) and provides pooled incidence rates to inform the consenting process. The difference in the incidence of unplanned reoperation rates (PF (3.7%) and FIN (5.8%)), which is not clinically significant, may favor PF as deformity and nail migration necessitating early removal is secondary to relative stability conferred by FIN (Figure 3(a)). Whereas, the difference in leg-lengths favoring FIN (PF (3%) and FIN (2.6%)) may be secondary to the anatomical reduction in PF leading to overgrowth. In our review, time to union is longer in the PF group (PF 10.6 vs FIN 8.9 weeks) which is not in keeping with published literature. This could be because studies reporting union were only referring to radiological parameters and primary healing in PF does not produce as much callus as a secondary process in FIN and may be harder to see. Furthermore, time to union is often influenced by the timing of follow-up appointments so this difference is unlikely to be clinically significant. We hypothesize that the lack of difference in some outcomes between the groups means that either there really is no difference or the patient numbers are not large enough to reliably show a difference.

## Strengths and limitations

The ROBINS-I and RoB 2 tools assessed the risk of bias for non-randomized and randomized interventional study designs, respectively. Using the ROBINS-I tool (Table 5), the quality overall of the research assessing surgical management of closed pediatric femoral diaphyseal fractures was very low. Reporting of definitions was inconsistent, and most studies were methodologically flawed, therefore, quantifying true risk in pooled analyses was challenging. This introduced a bias to real-world risk estimates and small sample sizes created wide confidence intervals in meta-analyses. As the risk of bias, predominantly from confounding within the observational, retrospective, non-randomized included studies was high, it was not possible to make robust treatment recommendations. Surgeon preference was an additional significant confounding factor. However, relative to research in this specific area, a pooled analysis of 805 fractures with minimal variance may be considered high quality.

## Conclusion

This analysis suggests that a pediatric femoral diaphyseal fracture treated with PF is associated with a low risk of soft tissue infection. However, there is no difference between PF and FIN groups with regards to malunion, unplanned reoperation and leg-length difference. Our work further informs consenting practice and highlights that the operative treatment of pediatric femoral shaft fracture remains an area of clinical equipoise. Importantly, this paper goes beyond just “advocating” for prospective trials, it provides a robust appraisal of the available literature and the statistical foundation to justify such a study. It also provides trialists with the necessary incidence information to design adequately powered prospective studies.

## Supplemental Material

sj-docx-2-cho-10.1177_18632521231190713 – Supplemental material for Plate fixation versus flexible intramedullary nails for management of closed femoral shaft fractures in the pediatric population: A systematic review and meta-analysis of the adverse outcomes Click here for additional data file. Supplemental material, sj-docx-2-cho-10.1177_18632521231190713 for Plate fixation versus flexible intramedullary nails for management of closed femoral shaft fractures in the pediatric population: A systematic review and meta-analysis of the adverse outcomes by Abhinav Singh, William Bierrum, Justin Wormald, Manoj Ramachandran, Gregory Firth and Deborah Eastwood in Journal of Children’s Orthopaedics

sj-pdf-1-cho-10.1177_18632521231190713 – Supplemental material for Plate fixation versus flexible intramedullary nails for management of closed femoral shaft fractures in the pediatric population: A systematic review and meta-analysis of the adverse outcomes Click here for additional data file. Supplemental material, sj-pdf-1-cho-10.1177_18632521231190713 for Plate fixation versus flexible intramedullary nails for management of closed femoral shaft fractures in the pediatric population: A systematic review and meta-analysis of the adverse outcomes by Abhinav Singh, William Bierrum, Justin Wormald, Manoj Ramachandran, Gregory Firth and Deborah Eastwood in Journal of Children’s Orthopaedics
